# Allele variation at the *Rdm3* locus conferring resistance to soybean southern stem canker

**DOI:** 10.1007/s00122-026-05381-9

**Published:** 2026-09-27

**Authors:** M. Habib Widyawan, James W. Buck, Zenglu Li

**Affiliations:** 1 Institute of Plant Breeding, Genetics, and Genomics, Department of Crop and Soil Sciences, University of Georgia, Athens, GA 30602 USA; 2 Department of Plant Pathology, University of Georgia, Griffin, GA 30223 USA

## Abstract

**Key message:**

Crockett and PI 398469 carry distinct resistance alleles at the *Rdm3* locus for soybean southern stem canker, supported by progeny analysis, sequence variation, candidate gene expression profiling, and allele transmission patterns.

**Abstract:**

Genetic resistance to southern stem canker (SSC) in soybean is predominantly conditioned by the *Rdm3* locus on chromosome (Chr) 14. Resistance at this locus has been identified in ‘Crockett’ and PI 398469; however, the allelic relationship between these lines remains unresolved. This study aimed to determine whether SSC resistance in Crockett and PI 398469 is governed by the same or distinct alleles at the *Rdm3* locus. Progeny analysis of a Crockett × PI 398469 recombinant inbred line (RIL) population identified recombinant lines with weaker resistance and broader phenotypic variation than either parent, indicating that recombination at the *Rdm3* locus disrupted favorable allele combinations and generated unexpected SSC resistance phenotypes. Consistent with this, sequence analysis of two priority candidate genes, *Glyma.14G023500* and *Glyma.14G022600*, revealed variation associated with resistance between Crockett and PI 398469, as well as among resistant and susceptible RILs. Expression profiling of these candidate genes further demonstrated genotype-dependent temporal patterns, suggesting distinct regulatory mechanisms underlying resistance conferred by *Rdm3* alleles from Crockett and PI 398469. In addition, the resistance alleles differed in their transmission behavior, with segregation distortion and underrepresentation of the Crockett allele observed across breeding populations and germplasm pools. Pedigree analysis further indicated an atypical origin of the Crockett *Rdm3* allele. These results demonstrate that Crockett and PI 398469 carry functionally distinct resistance alleles at *Rdm3*, confirming allelic variation at a major SSC resistance locus and providing insight into the effective deployment of *Rdm3* resistance in soybean breeding.

**Electronic supplementary material:**

The online version of this article (https://doi.org/10.1007/s00122-026-05381-9) contains supplementary material, which is available to authorized users.

## Introduction

Southern stem canker (SSC) in soybean, caused by the ascomycete fungus *Diaporthe aspalathi* (syn. *Diaporthe phaseolorum* var. *meridionalis*), has resulted in significant economic losses in the southern United States and South America (Wrather et al. 2018). Yield losses due to SSC range from 20 to 80%, depending on cultivar resistance and environmental conditions (Harville et al. 1986). Over the last two decades, SSC has caused an estimated $226 million in economic losses in the southern U.S. soybean production region (CPN 2025). Among the available management strategies, the use of genetically resistant cultivars is the most effective and sustainable approach for managing SSC (Backman et al. 1985; George et al. 2025).

Resistance to SSC in soybean is conferred by *Resistance to Diaporthe meridionalis* (*Rdm*) genes, a nomenclature established prior to the reclassification of the causal pathogen as *D. aspalathi*. To date, five *Rdm* genes (*Rdm1*-*Rdm5*) have been identified based on classic segregation analyses of F₂ populations (Tyler 1995) and differential reactions to pathogen isolates (Chiesa et al. 2009). Among these, *Rdm3* has consistently emerged as a major source of SSC resistance across multiple association panels, including subsets of the USDA soybean germplasm collection (Chang et al. 2016), Brazilian germplasm (Maldonado dos Santos et al. 2019), and elite southern breeding lines (Widyawan et al. 2026).

The *Rdm3* locus on Chr 14 was first identified in the resistant soybean cultivar Crockett using a classic genetic study (Bowers et al. 1993) and later identified in PI 398469 (Menke et al. 2023), while the *Rdm3* Tracy-M-type was recently identified as a major source of SSC resistance in elite southern soybean germplasm (Widyawan et al. 2026); these resistance sources had previously been assigned different resistance gene symbols based on segregation analyses of F_2_ populations (Bowers et al. 1993; Tyler 1995). Compared to other *Rdm* sources, *Rdm3*-bearing genotypes Tracy-M, Crockett, and PI 398469 have demonstrated resistance to a broad range of *D. aspalathi* isolates collected from the USA and South America (Campbell et al. 2017; Brumer et al. 2018; Mohan et al. 2023). These findings highlight the widespread presence and importance of the *Rdm3* resistance alleles in providing SSC resistance in soybean breeding programs.

Allelic variation within a resistance locus can provide valuable genetic resources for improving resistance durability. For example, allelic variants at the *Rpp4* locus (*Rpp4-a* and *Rpp4-b*) confer differential resistance to Asian soybean rust isolates (King et al. 2017). Similarly, the *rhg1* resistance allele from PI 437654 provides broader resistance to soybean cyst nematode (SCN) races than the PI 88788 resistance allele at the same locus (Brucker et al. 2005). Evidence from haplotype structure (Widyawan et al. 2026) and differential responses to *D. aspalathi* isolates (Brumer et al. 2018) suggests that Crockett and PI 398469 harbor distinct resistance alleles at the *Rdm3* locus. However, an allelism test has not yet been conducted to determine whether these sources carry identical or distinct resistance alleles at *Rdm3*. Resolving the allelic relationship at the *Rdm3* locus between Crockett and PI 398469 is critical for guiding effective deployment strategies and avoiding redundant use of the same resistance alleles in breeding programs. Therefore, this study aimed to resolve the allelic relationship between Crockett and PI 398469 at the *Rdm3* locus using progeny analysis, integrated sequence analysis, and candidate gene expression profiling.

## Materials and methods

### Plant materials

Development of the experimental population began with a cross between Crockett and PI 398469. Crockett is a maturity group (MG) VIII, SSC-resistant soybean cultivar derived from ‘Hampton 266A’ × PI 171451 which was developed by Texas A&M University in 1978 (Bowers 1990). Hampton 266A is a selection from Hampton, which originated from the cross of ‘Majos’ × ‘Lee’ and is susceptible to SSC. PI 171451 is an SSC-resistant plant introduction originated from Kanagawa, Japan. PI 398469 is an MG VI soybean accession collected from South Korea in 1975 and was identified as resistant to SSC through evaluation of the USDA Soybean Germplasm Collection (Tyler 1995).

The cross between Crockett and PI 398469 was made in the summer of 2022 at the J. Phil Campbell Sr. Research and Education Center in Watkinsville, GA. F_1_ plants were grown during the 2022–2023 winter season at the Illinois Crop Improvement Association (ICIA) farm in Puerto Rico. The resulting F_2_ population was grown in the summer of 2023 at the Iron Horse Farm (IHF) in Watkinsville, GA. Seeds harvested from F_2_ plants in fall 2023 were advanced to the F_3_ and F_4_ generations at the ICIA farm during the 2023–2024 winter season. In the 2024 growing season, F_5_ seeds were planted at IHF, and 290 individual plants were harvested to establish an F_5_-derived recombinant inbred line (RIL) population.

### DNA extraction and genotyping

Genomic DNA was extracted from RILs using a bulk of five seeds from each line (King et al. 2014). Two stainless steel BB pellets were added to each well, the plate was sealed with cap mats, and seeds were ground using a SPEX SamplePrep Geno/Grinder (SPEX SamplePrep, Metuchen, NJ, USA). DNA concentration and quality were assessed using a TECAN Infinite M1000 Pro plate reader (TECAN Group, Männedorf, Switzerland) and normalized to 10 ng μL⁻^1^.

Genotyping was performed using three kompetitive allele-specific PCR (KASP) SNP assays targeting *Rdm3* haplotypes: GSM1395, GSM933, and GSM1402 on Chr 14 (Widyawan et al. 2026). Markers used to genotype RIL population are described in Table 1. Briefly, SNP GSM1395 targets the common resistance haplotype at the *Rdm3* locus, whereas GSM933 and GSM1402 are specific to the PI 398469 and Crockett haplotypes, respectively. Primers for the KASP markers are listed in Supplementary Table 1. KASP genotyping of the RIL population was conducted following Pham et al. (2015). Endpoint fluorescence detection and allele calling were performed using a TECAN Infinite M1000 Pro plate reader.

**Table 1:** *Rdm3* haplotype classes and corresponding KASP marker genotypes in the Crockett × PI 398469 recombinant inbred line (RIL) population

Haplotype class	Description	Genotypes at *Rdm3*-defining markers^1^			*Rdm3* haplotype^2^
		GSM1395^A^	GSM933^B^	GSM1402^C^	
Crockett	RILs carrying the Crockett haplotype	C	G	T	CGT
PI 398469	RILs carrying the PI 398469 (P2) haplotype	C	T	C	CTC
Recombinant #1(R1)	Recombinants carrying *Rdm3* resistance haplotypes from Crockett and PI 398469	C	T	T	CTT
Recombinant #2 (R2)	Recombinants carrying *Rdm3* alternate haplotypes from Crockett and PI 398469	C	G	C	CGC
Susceptible (S)	Susceptible check (G81-2057)	T	G	C	TGC

^1^Bold text indicates the resistance haplotype at the *Rdm3* haplotype-defining markers

^2^*Rdm3* haplotypes ordered by GSM1395, GSM933, and GSM1402 KASP markers

^A^Targets the *Rdm3* common resistance haplotype on Chr 14 (1,630,331 bp; Wm82.a6)

^B^Targets the PI 398469 resistance haplotype on Chr 14 (1,684,178 bp; Wm82.a6)

^C^Targets the Crockett resistance haplotype on Chr 14 (1,698,329 bp; Wm82.a6)

### Evaluation of segregation distortion

In addition to the Crockett × PI 398469 RIL population, these KASP markers were used to genotype earlier generations of the Crockett × PI 398469 population (F_2_ and F_2:3_), as well as four independents mapping populations, to evaluate segregation distortion at the *Rdm3* locus across different genetic backgrounds. These populations included the G00-3213 × Tracy-M F_2:3_, Tracy-M × Crockett F_2:3_, G81-2057 × Crockett RIL, and G81-2057 × PI 398469 F_2:3_ and RIL populations. Segregation distortion was evaluated by comparing the observed and expected segregation ratios using χ2 tests*.*

### *Rdm3* haplotype selection and phenotyping

To phenotype Crockett × PI 398469 progenies, RILs were selected based on their *Rdm3* haplotype composition. Four haplotype classes at *Rdm3* were represented: (1) RILs carrying the Crockett haplotype (*Rdm3_Crockett*, CGT; *n* = 22), (2) RILs carrying PI 398469 haplotype (*Rdm3_PI 398469,* CTC; *n* = 20), (3) R1, recombinants carrying *Rdm3* resistance haplotypes from both Crockett and PI 398469 (CTT; *n* = 3); and (4) R2 recombinants carrying alternate *Rdm3* haplotypes from both Crockett and PI 398469 (CGC, *n* = 22) (Table 1).

SSC phenotyping of the selected RILs was conducted using a modified toothpick inoculation procedure described by Campbell et al. (2017) and Menke et al. (2023) at the University of Georgia (UGA) Plant Pathology Greenhouse in Griffin, GA. Six replications of the selected RILs, with two plants per line per replication, were evaluated for disease response using a randomized complete block design (RCBD). Sterile toothpicks grown on potato dextrose agar (PDA) inoculated with the *D. aspalathi* bulk isolate BL3-15, collected in Bledsoe, GA, were used for inoculation.

At the vegetative V3 growth stage, a sterile dissecting needle was used to create a small puncture wound between the cotyledon and unifoliate nodes. A toothpick colonized by *D. aspalathi* was inserted into the wound and sealed with petroleum jelly to facilitate infection. The population parents and susceptible checks, J77-339 and G81-2057, were included for comparison. Disease ratings were recorded when the susceptible checks began to develop symptoms, approximately three to four weeks after inoculation, using a 1–5 scale: 1 = no disease; 2 = cortical discoloration; 3 = visible stem canker or lesions; 4 = stem lesions with necrosis and interveinal chlorosis; and 5 = plant death.

### Statistical analysis

To evaluate differences in SSC disease ratings among RIL haplotype classes, analysis of variance (ANOVA) was conducted using a linear model in R. Haplotype class and replication were included as fixed effects. Significance of model terms was evaluated using ANOVA implemented in the CAR package (Fox and Weisberg 2019). When significant differences were detected, pairwise comparisons among haplotype classes were performed using Tukey-adjusted estimated marginal means implemented in the emmeans package at α = 0.05 (Lenth & Piaskowski 2025).

### Whole-genome resequencing (WGRS) and variant calling

Crockett, PI 398469, two resistant and two susceptible RILs derived from Crockett × PI 398469 selected based on greenhouse phenotyping, and Crockett’s parents (Hampton 266A and PI 171451) were sequenced at ~30× depth using Illumina NovaSeqX (150 bp paired-end reads). The selected Crockett × PI 398469 RILs are listed in Supplementary Table 2. Sequencing was performed at the Georgia Genomics and Bioinformatics Core (GGBC), University of Georgia (RRID: SCR_010994). Variants were called against the Williams 82 reference genome (version 6; Espina et al. 2024) using GATK v4.4.0.0 following Best Practices (DePristo et al. 2011; Van der Auwera et al. 2013).

### Candidate gene analysis

Candidate genes underlying the *Rdm3* locus were compiled from previous mapping studies in Crockett and PI 398469 (Menke et al. 2023). Gene ontology (GO) enrichment analysis was performed to assess the functional categories of these candidate genes using the SoyBase GO analysis tool (https://www.soybase.org/tools/analysis/go.html), following the approach described by Morales et al. (2013). To further characterize sequence variation among candidate genes, variant call data were used to reconstruct sample-specific nucleotide sequences at priority candidate gene regions by incorporating SNPs and short insertions/deletions (indels) from the variant call format (VCF) into the Williams 82 reference genome (version 6), generating consensus FASTA sequences for each line. These sequences were imported into Geneious Prime v2023.0.1 (www.geneious.com) for nucleotide alignment. Coding regions were translated into amino acid sequences and aligned separately in Geneious Prime.

### Microscopic imaging analysis of *D. aspalathi* disease development.

Microscopy imaging analysis was conducted to characterize *D. aspalathi* colonization and disease progression in soybean stems to determine appropriate tissue sampling time points for downstream gene expression analysis. The SSC-resistant soybean lines Crockett and PI 398469, along with the SSC-susceptible line G81-2057, were inoculated with *D. aspalathi* as described above. Thin longitudinal stem sections were collected from inoculated plants at 3, 6, 9, 12, 24, and 72 h post inoculation (HPI), as well as 7 and 14 d post inoculation (DPI). For each time point, stem sections were prepared using a sterile razor blade and mounted on clean microscope slides.

Fungal structures were visualized using calcofluor white staining (Sigma-Aldrich, St. Louis, MO, USA). A drop of staining solution containing 10% potassium hydroxide and calcofluor white was applied to each stem section, followed by incubation in the dark for 1 min. A coverslip was placed over the sample and gently pressed to remove excess liquid. Remaining stain was evaporated by briefly passing the edge of the coverslip over an alcohol flame. Slides were sealed with clear nail polish and stored in the dark prior to imaging. Microscopic observations were performed using an Olympus BX40 microscope (Olympus Corporation, Tokyo, Japan) equipped with an Omega DAPI filter. Observations of fungal penetration, hyphal expansion, and host tissue responses were used to guide the selection of sampling time points for subsequent quantitative real-time PCR (qRT-PCR) analysis.

### Pathogen inoculation and tissue sampling

Based on the analysis, four sampling time points were selected for gene expression analysis: 9 h, 24 h, 72 h and 7 d post inoculation. Seven soybean genotypes were included in this experiment: Crockett, PI 398469, the susceptible line G81-2057, and two resistant and susceptible RILs derived from Crockett and PI 398469 in a G81-2057 background (Supplementary Table 3). G19-2950 (resistant) and G19-3059 (susceptible) are RILs from G81-2057 × Crockett, while G19-1860 (resistant) and G19-1857 (susceptible) are from G81-2057 × PI 398469. The RILs derived from the G81-2057 × Crockett and G81-2057 × PI 398469 crosses were part of the RIL populations used to map the *Rdm3* locus from Crockett and PI 398469 (Menke et al. 2023). The experiment was arranged in a RCBD with three biological replicates, each consisting of two plants per genotype. Plants were grown at the UGA Plant Growth Facilities (PGF), Athens, GA, under controlled conditions in a growth chamber with day/night temperatures of 28 and 21 °C, respectively, and a 16 h light / 8 h dark photoperiod.

Inoculation was performed using the *D. aspalathi* isolate BL3-15. In addition, mock-inoculated controls were prepared using sterile toothpicks submerged in double-distilled water. Stem tissue samples (~ 3 cm) were collected using sterile scissors from the region between the cotyledon and unifoliate nodes, including the inoculation site. Samples were immediately flash-frozen in liquid nitrogen and stored at −80 °C until further processing. Frozen stem tissues were ground to a fine powder in liquid nitrogen using a mortar and pestle and stored at −80 °C prior to RNA extraction.

#### RNA extraction and quantitative real-time PCR (qPCR) analysis

Total RNA was extracted from approximately 300 mg of ground stem tissue/sample using a CTAB-based method (Gambino et al. 2008; Joly, 2024). When necessary, additional RNA cleanup was performed using the RNA Clean & Concentrator-25 kit (Zymo Research, Irvine, CA, USA). Genomic DNA was removed using the Zymo DNase I Set (Zymo Research) following the manufacturer’s instructions. RNA quality was assessed for all samples using a TECAN Infinite M1000 (TECAN Group Ltd. Männedorf, Switzerland), and for the samples by electrophoresis on a 2% agarose gel. RNA concentration was quantified using the Qubit™ RNA Broad Range (BR) Assay Kit on a Qubit™ Fluorometer (Thermo Fisher Scientific, Waltham, MA, USA).

RNA concentration was normalized to 200 ng µL⁻1 prior to cDNA synthesis. First-strand cDNA synthesis was conducted using the iScript™ cDNA Synthesis Kit (Bio-Rad Laboratories, Hercules, CA, USA) in a 20 µL reaction volume. qRT-PCR was performed in a 10 µL reaction volume using SsoAdvanced™ SYBR® Green Supermix on a CFX Opus 384 RT PCR System (Bio-Rad Laboratories, Hercules, CA, USA). Each sample was analyzed in three technical replicates using gene-specific primers (Supplementary Table 1). Primer efficiencies were validated and confirmed to be within an acceptable range (90–110%) (Bustin et al 2009). Gene expression analysis was conducted using the comparative Ct method (2⁻ΔΔCt) (Livak & Schmittgen 2001). For each sample, the ΔCt value was calculated by subtracting the Ct value of the internal reference gene from that of the target gene. The ΔΔCt was then obtained by subtracting the ΔCt of the mock-inoculated control from the ΔCt of the *D. aspalathi*–inoculated treatment. Relative gene expression levels were calculated using the 2⁻ΔΔCt method and expressed as log₂ fold change (log₂FC) relative to the control condition. Statistical analyses were performed on ΔCt values, and differences between inoculated and mock treatments were evaluated using two-sample t-tests for each genotype and time point. The metalloprotease gene *Cons7* on Chr 3 (Libault et al. 2008) was used as the internal reference gene to normalize Ct values for both inoculated and mock-inoculated samples.

#### Tracing the origin of Rdm3 locus from Crockett

To trace the origin of the *Rdm3* locus in Crockett, pedigree data were obtained from SoyBase parentage and pedigree reports (www.soybase.org). *Rdm3* haplotypes of Crockett and its parental and ancestral lines were retrieved from SoySNP50K genotype data of the USDA Soybean Germplasm Collection (Song et al. 2015). For lines not included in Song et al. (2015), *Rdm3* haplotypes were determined using KASP markers targeting the *Rdm3* locus. SSC phenotype data for this lineage, when available, were compiled from UGA greenhouse screenings, published studies (Chang et al. 2016; Maldonado dos Santos et al. 2019), and the USDA GRIN database. To evaluate potential structural variation (SV) underlying the *Rdm3* locus within the Crockett lineage, whole-genome resequencing (WGRS) data from Crockett, Hampton 266A, and PI 171451 were analyzed using Manta (Chen et al. 2016) and DELLY (Rausch et al. 2012) with default parameters to detect structural variants, including deletions, insertions, and breakpoints.

## Results

### Segregation of marker loci in the RIL population

Prior to phenotyping, RILs were selected based on their *Rdm3* haplotypes to enable targeted evaluation of allelic effects at the *Rdm3* locus. Genotyping revealed four haplotypes: Crockett-type, PI 398469-type, Recombinant #1 (R1), and Recombinant #2 (R2), which occurred at different frequencies. Both parental types were present at higher frequencies (33.4% and 46.1% for Crockett and PI 398469, respectively) than the recombinant types. R1 occurred at a very low frequency (1%) compared to R2 (19%).

To further examine the observed haplotype distribution, RIL genotypes at GSM933 and GSM1402 were analyzed individually to test for segregation distortion at PI 398469 and Crockett haplotype markers, respectively (Supplementary Table 4). Significant segregation distortion was detected at GSM1402, with the Crockett resistance allele being underrepresented (χ2 = 29.8, P < 0.0001). In contrast, no segregation distortion was observed for the PI 398469-targeting marker GSM933 (χ2 = 1.5, P = 0.22). A similar pattern of significant segregation distortion and underrepresentation of the Crockett resistance allele at GSM1402 was also observed in earlier generations of the Crockett × PI 398469 pedigree (P < 0.0001), as well as in two additional populations derived from crosses of Crockett with Tracy-M (*Rdm3*) and G81-2057 (susceptible) (P < 0.01 and P = 0.005, respectively). (Table 2).

**Table 2:** Allele frequencies and chi-square tests for segregation distortion at the *Rdm3*-associated markers GSM933 and GSM1402

Marker	Haplotype	Expected ratio^1^ (Res:Sus)	Observed ratio^1^ (Res:Sus)	${\chi }^{2}$	P value	Segregation pattern
GSM933	*Rdm3_PI 398469*	142:142	134:150	1.5	0.22	No distortion
GSM1402	*Rdm3_Crockett*	142:142	96:188	29.8	<.0001	Significant distortion

^1^Res = resistance allele; Sus = susceptible allele

Analysis of GSM1395 genotypes, which targets the same SSC resistance marker allele shared among known *Rdm3* sources (Tracy-M, Crockett, and PI 398469), revealed a distinct segregation pattern in populations derived from non-Crockett sources, with no segregation distortion detected in Tracy-M- and PI 398469-derived populations (P = 1 and P = 0.68, respectively) (Supplementary Table 4). However, consistent with the distortion observed at the GSM1402 locus, significant segregation distortion at the GSM1395 locus was also detected in the G81-2057 × Crockett RIL population (χ2 = 4.96, P < 0.05).

### Phenotypes of selected RILs

RILs carrying the Crockett and PI 398469-types had mean disease ratings of 1.2 and 1.3, respectively, while R1 haplotype had a rating of 1.2. Based on Tukey’s HSD test (α = 0.05), these three haplotypes are clustered to the same resistance class group as expected based on their allele composition at the *Rdm3* locus (Table 3). In contrast, RILs with the R2 haplotype exhibited a higher mean of SSC disease rating (mean = 2.0) and formed a distinct group with a reduced level of resistance (P < 0.05). This haplotype also displayed a broader range of individual plant SSC ratings (1.0 to 5.0) compared to other haplotype classes, indicating greater phenotypic variability.

**Table 3:** Southern stem canker (SSC) disease ratings of selected recombinant inbred lines (RILs) derived from Crockett × PI 398469 population evaluated using *Diaporthe aspalathi* isolate BL3-15, classified by *Rdm3* haplotypes

RIL haplotype class	*Rdm3* Haplotype^1^	SSC Rating^2^ (mean ± SD)	Number of RILs^3^ (*n*)	Min-Max^4^
Crockett-type	CGT	1.21 ± 0.16^a^	21	1.00–1.58
PI 398469-type	CTC	1.28 ± 0.18^a^	20	1.00–1.67
Recombinant #1 (R1)	CTT	1.25 ± 0.17^a^	3	1.08–1.42
Recombinant #2 (R2)	CGC	1.98 ± 0.42^b^	22	1.33–2.83
Susceptible Check (G81-2057)	TGC	3.92 ± 0.27^c^	–	–

^1^*Rdm3* haplotype ordered by GSM1395 (common resistance haplotype at the *Rdm3*), GSM933 (resistance allele for *Rdm3_PI 398469*), and GSM1402 (resistance allele for *Rdm3_Crockett]*) KASP markers

^2^1 (resistant) to 5 (susceptible) scale. Values represent mean ± SD. Means followed by different letters are significantly different based on Tukey’s Honestly Significant Differences (HSD) (α = 0.05)

^3^Represents the number of recombinant inbred lines (RILs) included in each haplotype class. Mean SSC ratings were calculated from RIL means, with each RIL evaluated using two plants per replication across six experimental replications

^4^Represents the minimum and maximum mean SSC ratings among recombinant inbred lines (RILs) within each haplotype class

**Fig. 1:**
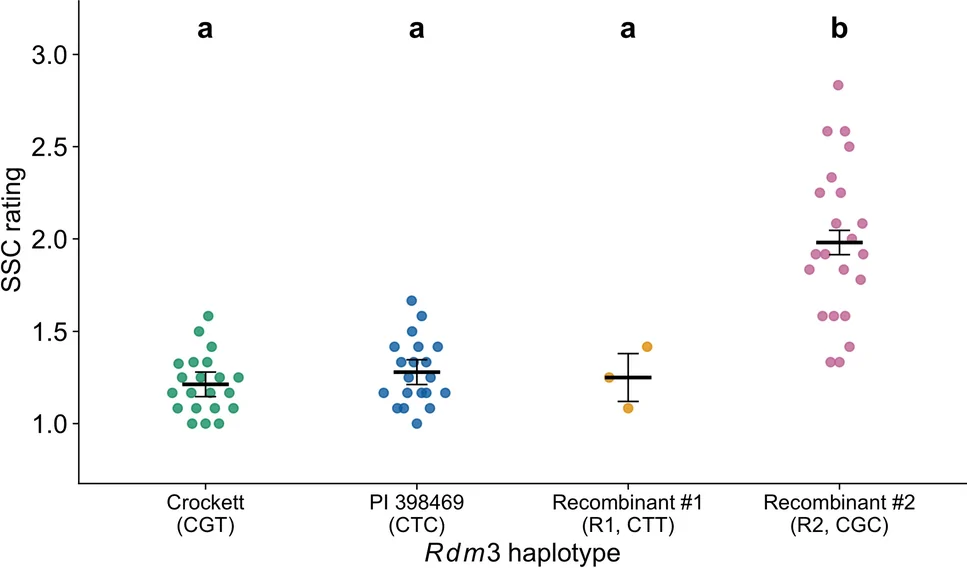
Distribution of mean southern stem canker (SSC) disease ratings (BLUEs) of selected recombinant inbred lines (RILs) from the Crockett × PI 398469 population, grouped by *Rdm3* haplotypes defined using KASP markers. Each point represents the BLUE of an individual RIL. Horizontal bars indicate the haplotype mean ± SE. Colors represent distinct *Rdm3* haplotype groups. Means followed by different letters differ significantly according to Tukey’s honestly significant difference (HSD) test (α = 0.05)

The susceptible check, G81-2057, displayed the highest mean of SSC disease ratings (3.9) and constituted a separate susceptibility class from both the parental and recombinant haplotypes (P < 0.05). Visualization of SSC disease ratings in the Crockett × PI 398469 RIL population further illustrates differences in phenotypic distributions among the selected *Rdm3* haplotypes (Fig. 1). R1 RILs exhibited disease ratings (mean = 1.2) and ranges similar (min = 1.0, max = 1.4) to those observed in the parental haplotypes, whereas R2 RILs showed a broader distribution of SSC responses (mean = 2.0, min = 1.3, and max = 2.8).

### Candidate gene analysis

**Fig. 2:**
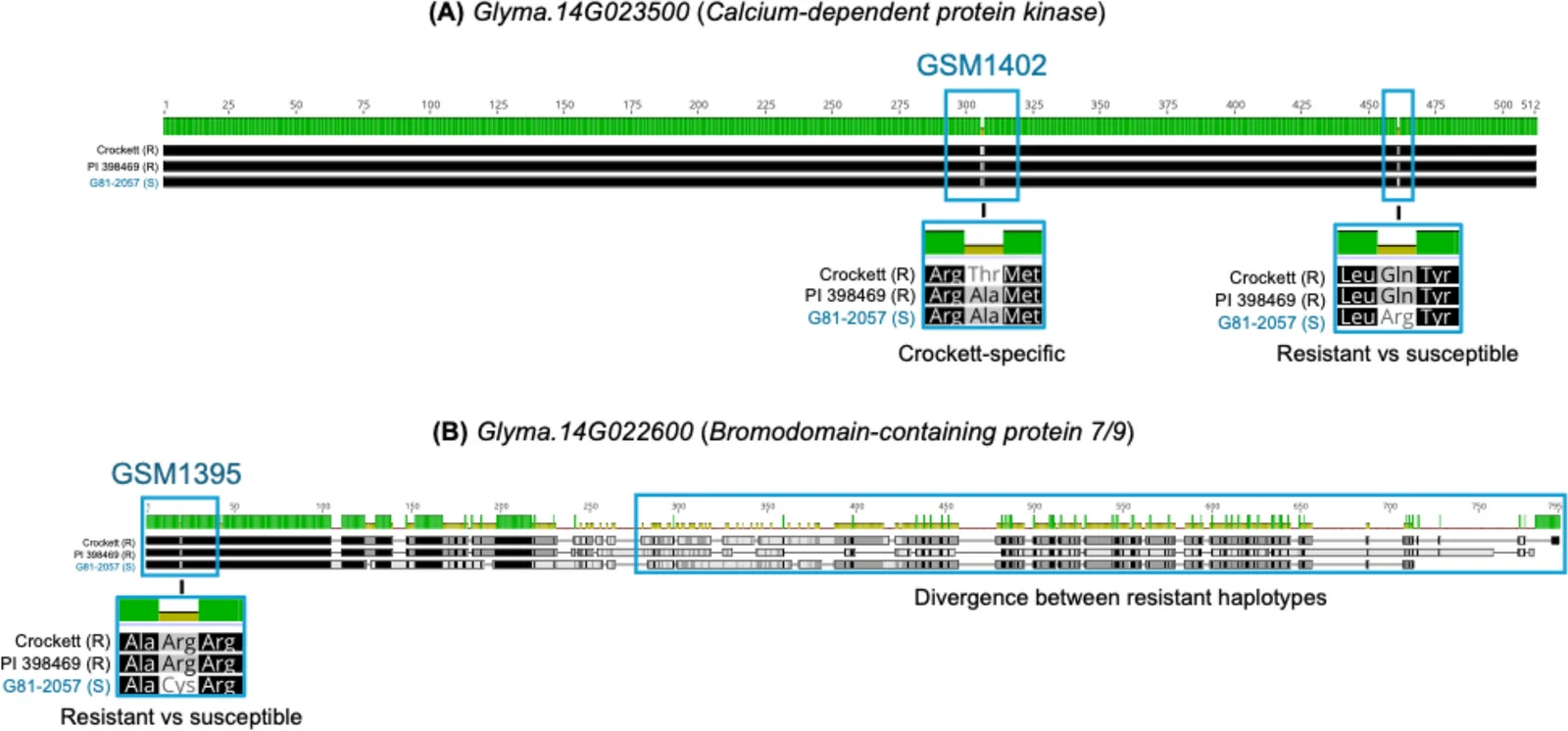
Amino acid alignment of candidate genes underlying SSC resistance at the *Rdm3* locus. (A) *Glyma.14G023500;* (B) *Glyma.14G022600*. Informative regions, including amino acid substitutions and haplotype divergence between resistant lines, are highlighted by blue boxes and annotated

Eighteen candidate genes within the *Rdm3* locus, previously identified through genetic mapping (Menke et al. 2023), were subjected to GO enrichment analysis. Significant enrichment of kinase- and signaling-related functions was observed (P < 0.01). Among the prioritized candidate genes, *Glyma.14G023500*, encodes a calcium-dependent protein kinase (CDPK) involved in protein phosphorylation and signal transduction. The KASP marker GSM1402, which defines the *Rdm3_Crockett* type, is located within this gene and corresponds to a missense substitution (threonine to alanine) specific to Crockett (Fig. 2A). An additional SNP identified from WGRS results in another amino acid substitution (glycine to arginine) that differentiates Crockett, PI 398469, and the susceptible G81-2057, indicating distinct amino acid profiles among these genotypes at this candidate gene.

In addition, GSM1395 and GSM975, consistently identified as tightly linked markers to *Rdm3*, are located within *Glyma.14G022600*, encoding a bromodomain-containing protein. The SNP targeted by GSM1395 results in an arginine-to-cysteine substitution (Fig. 2B), and sequence alignment revealed substantial divergence between Crockett and PI 398469 (41% similarity), indicating haplotype differentiation at this locus. While positional evidence supports *Glyma.14G022600* as a candidate gene, functional enrichment and haplotype-defining marker evidence highlight *Glyma.14G023500* as a promising candidate underlying *Rdm3*-associated resistance. These genes were therefore selected for further expression analysis.

### Sequence variation in candidate genes at the *Rdm3* locus in selected RILs

**Fig. 3:**
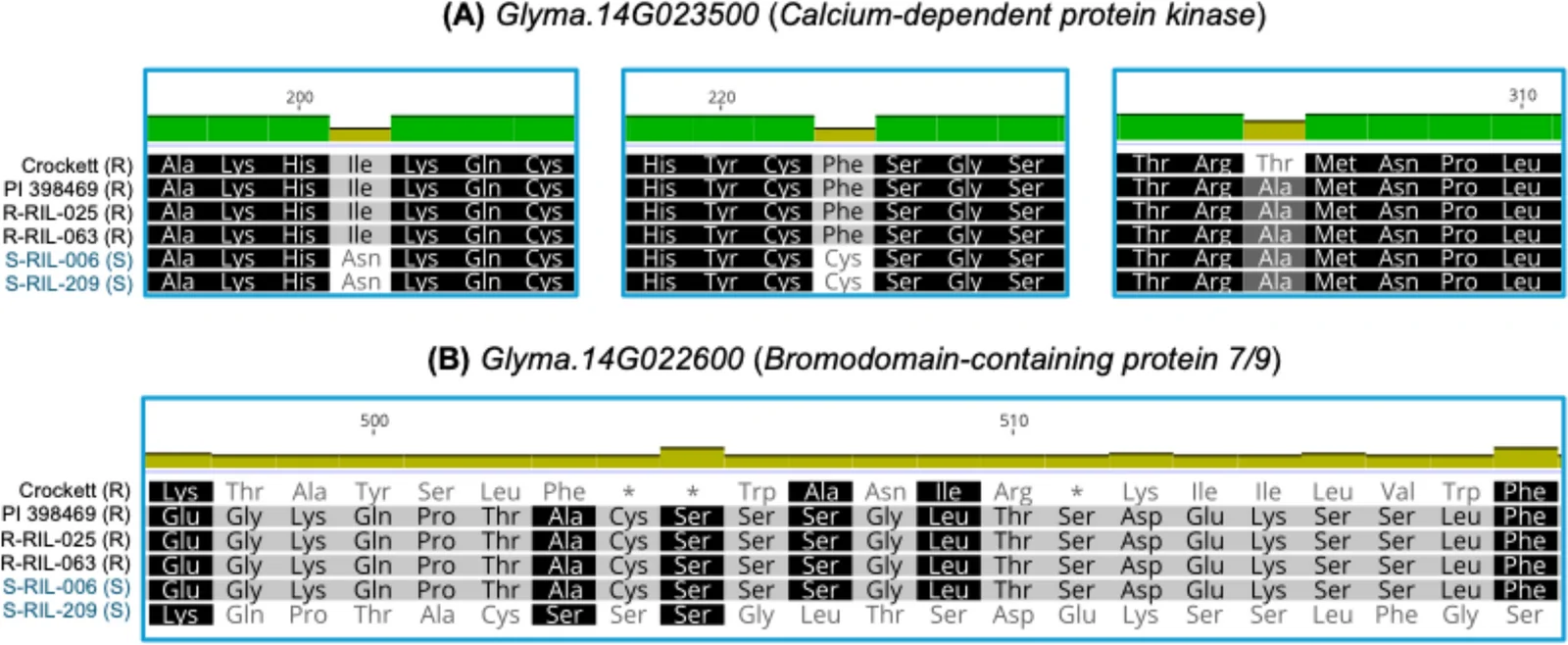
Amino acid alignment of *Rdm3* candidate genes in Crockett, PI 398469, Crockett × PI 398469-derived resistant and susceptible RILs: **A** *Glyma.14G023500* and B *Glyma.14G022600*

To investigate variable phenotypes observed in the R2 haplotype class, two resistant and two susceptible RILs from the Crockett × PI 398469 population were sequenced to assess nucleotide and amino acid variation in *Glyma.14G023500* and *Glyma.14G022600*. *Glyma.14G023500* exhibited >99% amino acid similarity across selected Crockett × PI 398469 RILs regardless of resistance status; however, two additional SNPs resulting in amino acid substitutions were identified that were specific to susceptible RILs (Fig. 3A). In contrast, resistant RILs showed the same sequence and amino acid profiles to PI 398469, differing only from Crockett by a single amino acid substitution (Fig. 3A). In *Glyma.14G022600*, PI 398469 and both resistant and susceptible RILs shared >99% sequence similarity, whereas Crockett exhibited substantial divergence (~ 16% similarity) (Fig. 3B), indicating haplotype differentiation at this locus. In contrast to *Glyma.14G023500*, the observed amino acid variation in *Glyma.14G022600* was not associated with resistance status among the RILs.

### Expression profiling of candidate genes in parental lines and RILs

**Fig. 4:**
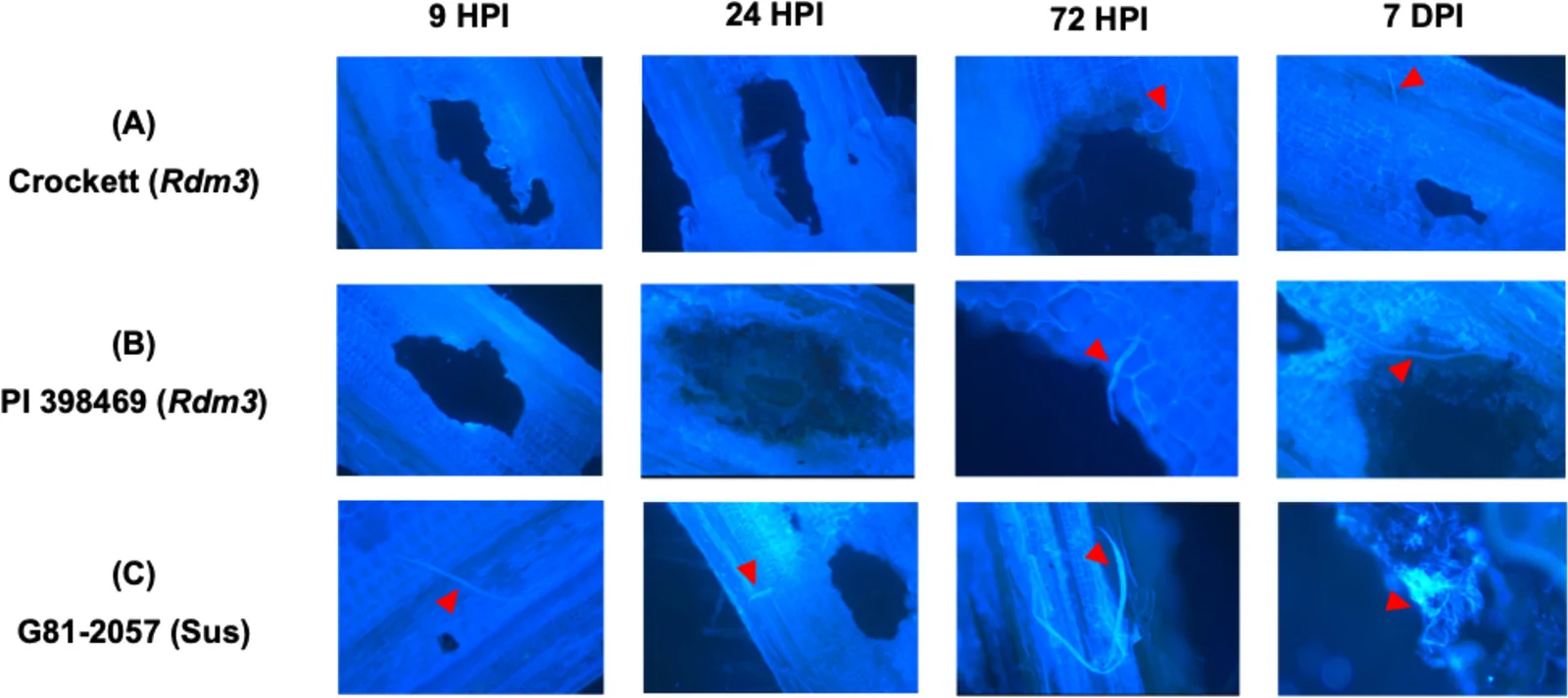
Microscopic visualization of *D. aspalathi* colonization during southern stem canker development. **A** Crockett (resistant); **B** PI 398469 (resistant); and **C** G81-2057 (Susceptible). Red arrowheads indicate *D. aspalathi* hyphae

Microscopy using calcofluor white staining revealed early hyphal growth in the susceptible line G81-2057 as early as 9 HPI, whereas in resistant lines (Crockett and PI 398469), hyphal expansion remained restricted despite detectable colonization by 72 HPI (Fig. 4). Based on these observations, four time points (9, 24, and 72 HPI, and 7 DPI) were selected for expression analysis.

**Fig. 5:**
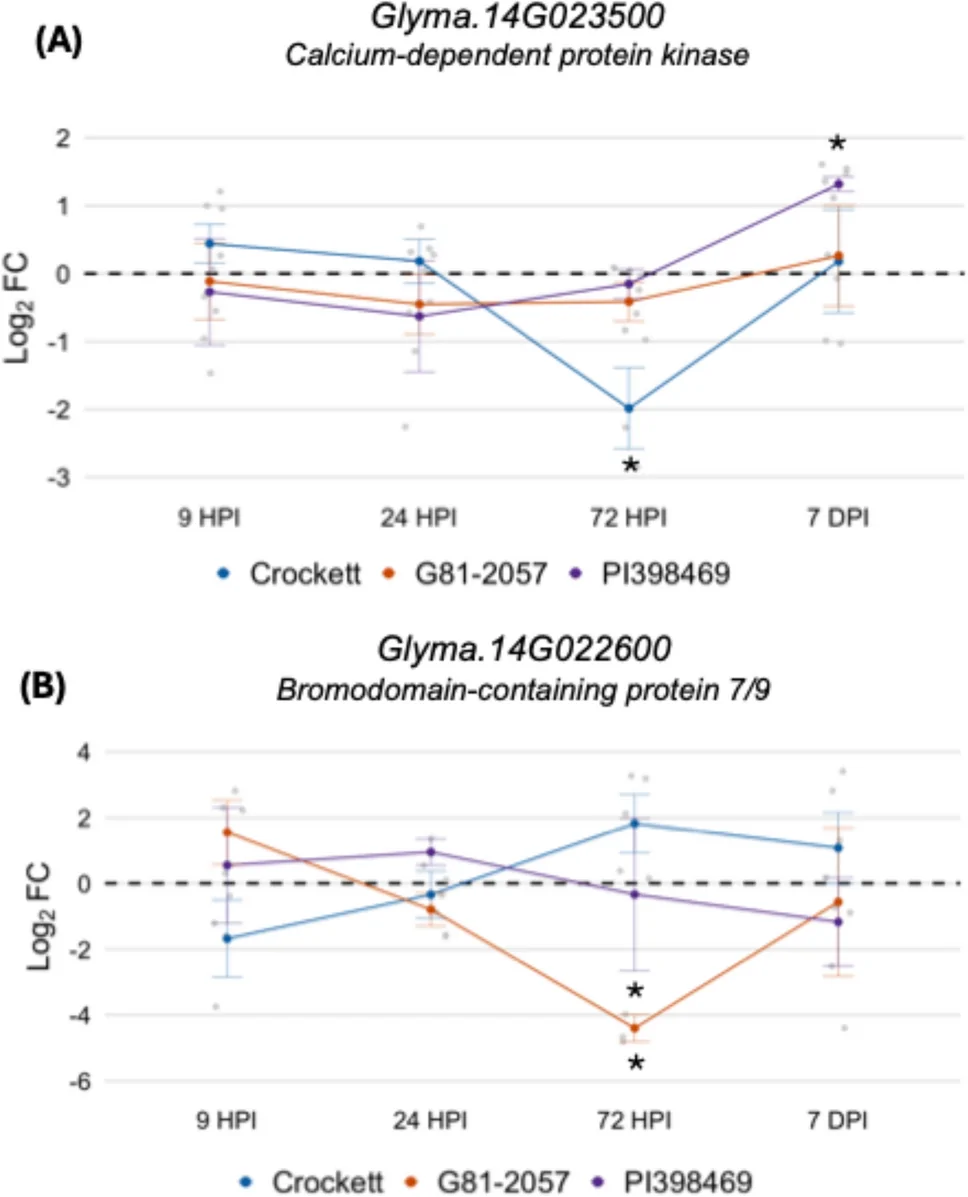
Expression profiles of candidate genes at the *Rdm3* locus following *D. aspalathi* inoculation in Crockett (*Rdm3_Crockett*), PI 398469 (*Rdm3_PI 398469*), and G81-2057 (susceptible). **A** Relative expression of *Glyma.14G023500*; **B** Relative expression of *Glyma.14G022600.* Asterisks indicate significant differences between inoculated and mock treatments (*P* < 0.05)

Expression profiling of *Glyma.14G023500* showed distinct genotype-dependent patterns (Fig. 5A). At 72 HPI, Crockett exhibited strong repression relative to mock (P < 0.05), coinciding with active colonization. In contrast, PI 398469, another resistant source, displayed significant upregulation at 7 DPI (P < 0.05), whereas expression in the susceptible G81-2057 remained near baseline across all time points. *Glyma.14G022600* also exhibited genotype-specific responses (Fig. 5B). At 9 HPI, expression was suppressed in Crockett but upregulated in G81-2057 (P < 0.05), while remaining stable in PI 398469. By 72 HPI, expression diverged further, with strong repression in G81-2057 and sustained expression in resistant lines, followed by convergence to baseline by 7 DPI.

**Fig. 6:**
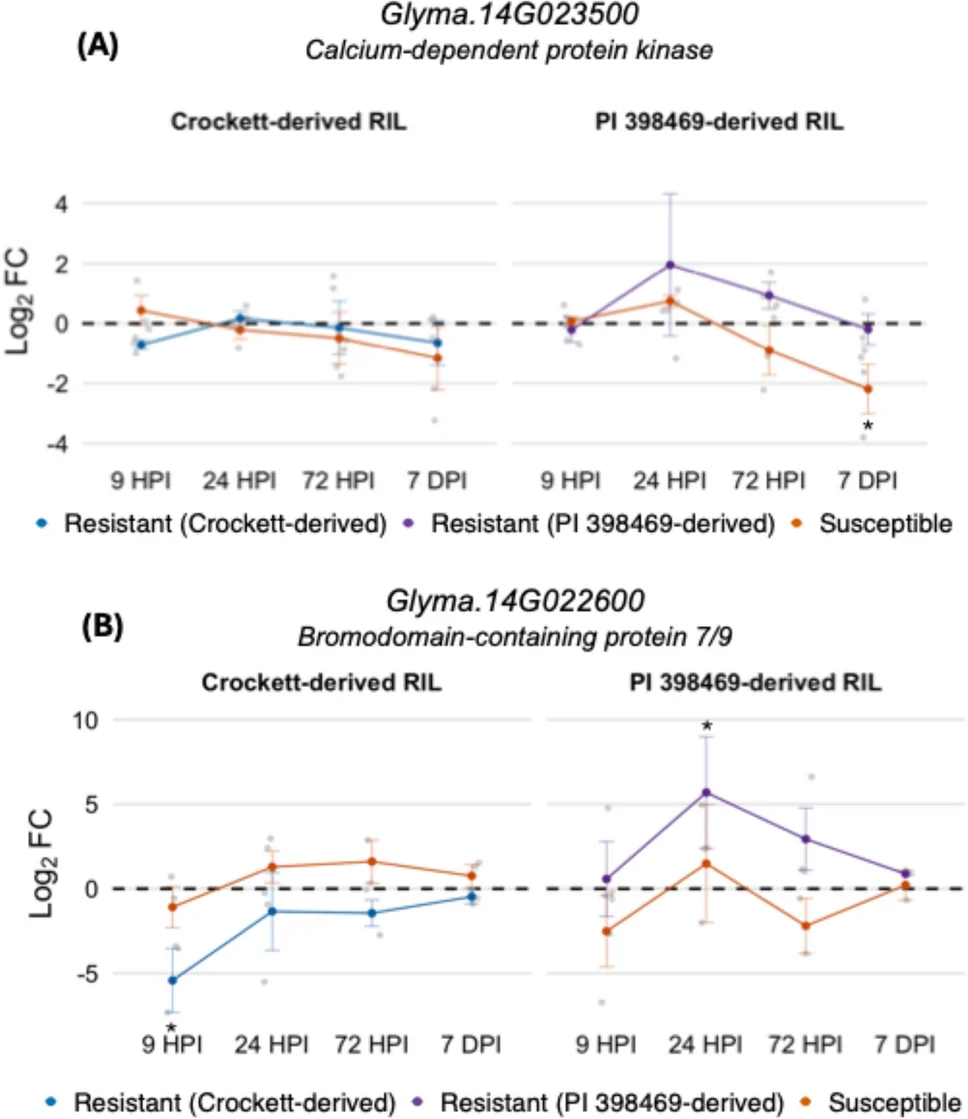
Expression profiles of candidate genes at the *Rdm3* locus following *D. aspalathi* inoculation in Crockett and PI 398469-derived resistant and susceptible RILs in G81-2057 background. **A** Relative expression of *Glyma.14G023500*; **B** Relative expression of *Glyma.14G022600.* Asterisks indicate significant differences between inoculated and mock treatments (P < 0.05)

Expression patterns of *Glyma.14G023500* in the RIL population were consistent with those observed in the parental trends (Fig. 6A). In the G81-2057 × Crockett population, resistant RILs showed early repression at 9 HPI, whereas susceptible RILs exhibited slight upregulation. In contrast, in the G81-2057 × PI 398469 population, *Glyma.14G023500* expression showed minimal early differences, with divergence emerging at 7 DPI, where susceptible RILs exhibited significant downregulation (P < 0.05). For *Glyma.14G022600*, early expression patterns in RILs similarly reflected parental responses (Fig. 6B), followed by convergence toward baseline at later stages. Notably, susceptible RILs from the PI 398469-derived population showed marked repression at 72 HPI.

### Pedigree analysis of the *Rdm3_Crockett* haplotype

**Fig. 7:**
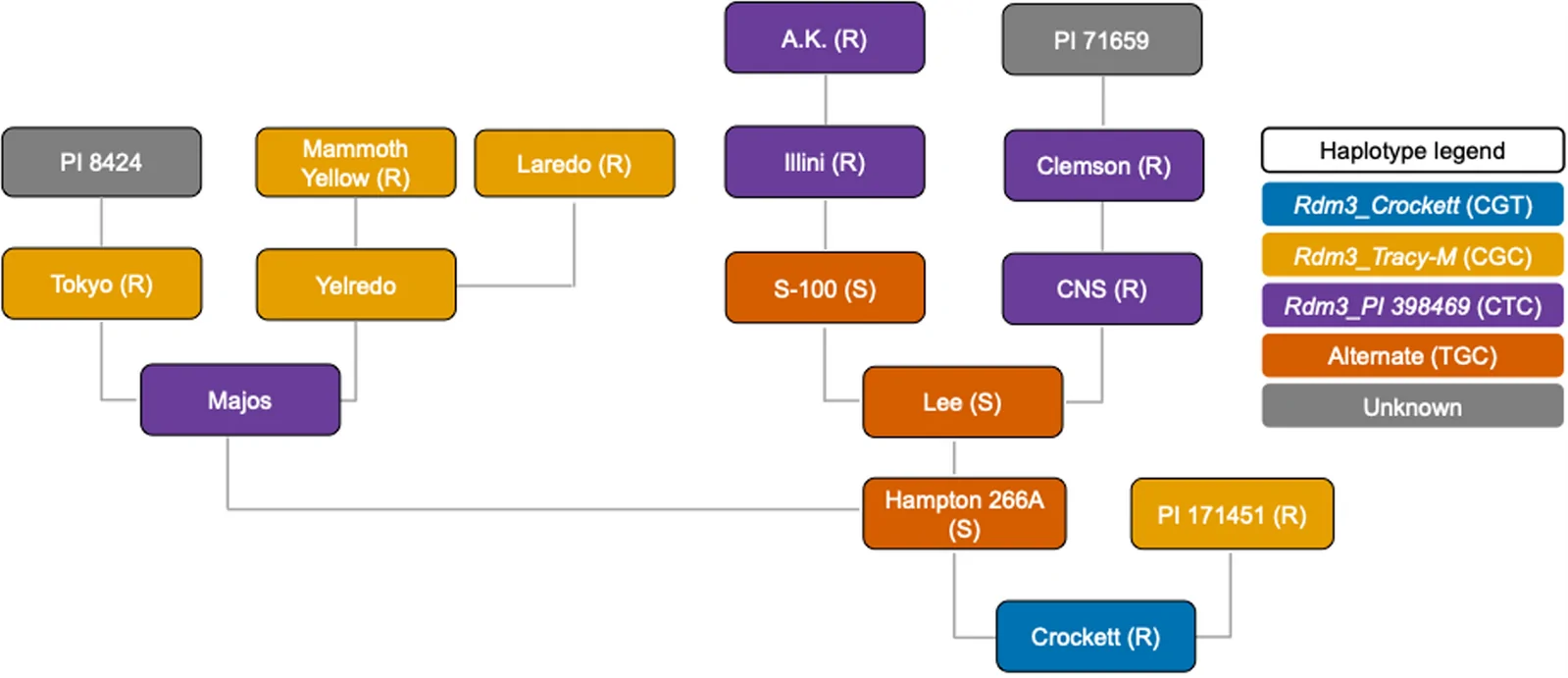
Pedigree analysis of the *Rdm3* haplotype in Crockett and its ancestral lines. Letters in parentheses indicate reported SSC resistance status: R = resistant and S = susceptible; resistance status is unknown for lines without a designation. *Rdm3* haplotypes are ordered by KASP markers GSM1395, GSM933, and GSM1402

The rarity of the *Rdm3_Crockett* type and underrepresentation of its resistance allele in breeding populations prompted investigation into its origin. Pedigree analysis using SoySNP50K and KASP genotyping showed that neither of Crockett’s parents (Hampton 266A and PI 171451) nor its documented grandparents carried the *Rdm3_Crockett* type (CGT) (Fig. 7). Hampton 266A and its ancestor Lee are both susceptible and carry an alternate *Rdm3* (TGC) not associated with known resistance, whereas PI 171451 is resistant and carries the *Rdm3_Tracy-M* type (CGC), indicating that the *Rdm3* Crockett-type cannot be explained by its reported pedigree.

**Fig. 8:**
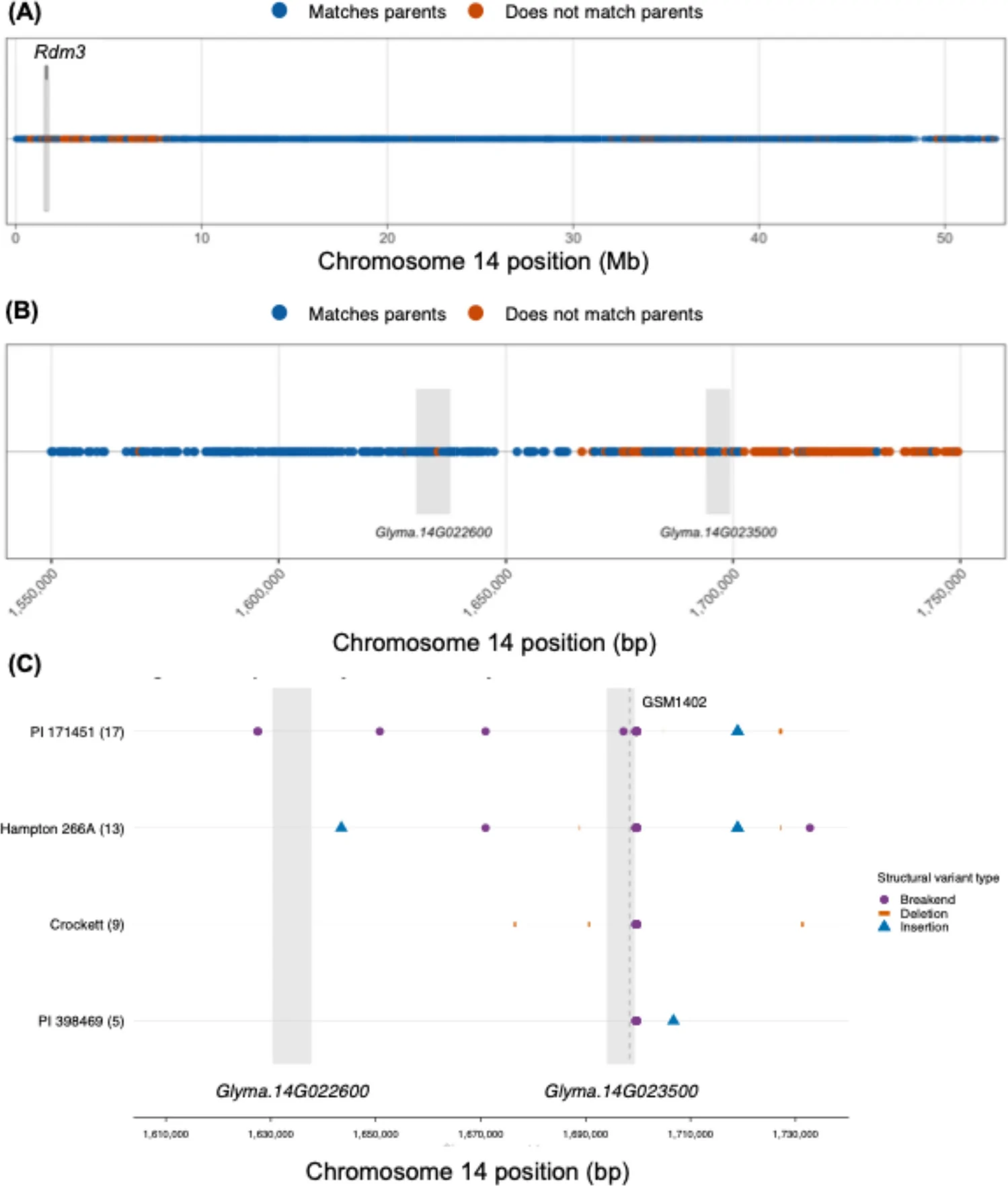
Evidence of non-Mendelian inheritance and structural variation at the *Rdm3* locus in Crockett. **A** Parental-offspring genotype matching on Chr 14 for Crockett and its parental lines Hampton 266A and PI 171451; **B** Zoomed view within the *Rdm3* interval on Chr 14; **C** Structural variation detected within the *Rdm3* interval across Crockett, its parental lines, and PI 398469

To further investigate, WGRS data were examined for Crockett and its parents, Hampton 266A and PI 171451. On Chr 14, 28% of Crockett SNP alleles were inconsistent with both parents, with mismatches concentrated in a ~7.5 Mb region encompassing the *Rdm3* locus (Fig. 8A). These discrepancies were enriched within candidate genes *Glyma.14G023500* and *Glyma.14G022600* (Fig. 8B), suggesting a non-random pattern unlikely to be explained by genotyping error. Structural variation analysis revealed elevated genomic complexity in this region. Hampton 266A and PI 171451 exhibited more structural variants (13 and 17, respectively) than Crockett (9), while PI 398469 showed the fewest (5). Structural variation was dominated by breakend (BND) signals, indicating complex rearrangements within the *Rdm3* interval (Fig. 8C).

## Discussion

### Determination of *Rdm3* resistance alleles from Crockett and PI 398469

Menke et al. (2023) identified SSC resistance in Crockett and PI 398469 as controlled by the *Rdm3* locus on Chr 14, with evidence suggesting distinct alleles based on haplotype variation (Widyawan et al. 2026) and differential responses to pathogen isolates (Brumer et al. 2018). Although both sources confer broad resistance to *D. aspalathi*, they respond differently to Brazilian isolates, with CMES 497 and CMES 480 causing greater susceptibility in PI 398469 (Brumer et al. 2018).

In this study, we demonstrated through progeny analysis, sequence variation, gene expression profiling, and allele transmission patterns that Crockett and PI 398469 carry distinct resistance alleles at *Rdm3*. Recombinant inbred lines (RILs) from Crockett × PI 398469 exhibited unexpected phenotypes, including significantly reduced resistance and increased variability, when carrying non-parental haplotype combinations, whereas RILs retaining parental or combined resistance haplotypes maintained stable resistance.

Sequence analysis of *Glyma.14G023500* and *Glyma.14G022600* identified amino acid variation between Crockett and PI 398469, supporting allelic differentiation. These genes were prioritized based on GO enrichment and positional evidence from previous mapping studies (Menke et al. 2023). Similar associations between amino acid variation and resistance have been reported at the flax *L* locus and rice *Ptr* locus (Ellis et al. 2007; Xiao et al. 2024). Among these candidates, *Glyma.14G023500* exhibited more consistent resistance-associated variation, with susceptible RILs carrying two amino acid substitutions within the protein kinase domain that may affect calcium-dependent signaling (Valmonte et al. 2014). In contrast, variation in *Glyma.14G022600* was less clearly associated with resistance status.

Recombination within resistance loci can generate novel allelic combinations and phenotypic variation (Kover & Caicedo 2001; Leister 2004; Johnston 2024). Thus, the unexpected SSC phenotypes observed among recombinants, together with the distinct amino acid profiles identified in susceptible RILs from the Crockett × PI 398469 population, support the presence of functionally distinct *Rdm3* alleles. Similar recombination-driven disruption has been reported at the flax *L* locus, where chimeric alleles resulted in susceptibility despite resistant parents (Ellis et al. 2007).

Notably, recombinant haplotypes were observed within an approximately 68-kb interval spanning the *Rdm3* haplotype-defining markers, representing about 20% of the population. Given the relatively short physical distance separating these markers, this observation suggests that crossover events occurred frequently within the *Rdm3* interval. Although this interval is physically small, recombination is not uniformly distributed across plant genomes. In particular, disease resistance loci often exhibit heterogeneous recombination landscapes, with crossover frequency influenced by local genomic architecture and sequence divergence (Choi et al. 2016; Serra et al. 2018). Therefore, the elevated recombination observed at *Rdm3* may reflect unique genomic features of this region, although additional studies will be required to determine the underlying molecular mechanisms.

Gene expression analysis further supported allelic differentiation from sequence analysis. *Glyma.14G023500*, encoding a calcium-dependent protein kinase (CDPK), showed genotype-specific temporal expression, with Crockett exhibiting early induction followed by repression, and PI 398469 showing delayed induction. Given that CDPK loss-of-function increases susceptibility (Coca & Segundo, 2010), these contrasting patterns suggest allele-specific regulation of calcium-mediated immune signaling.

*Glyma.14G022600*, encoding a bromodomain-containing protein, also showed genotype-dependent expression, with opposing early responses in resistant genotypes and strong repression in the susceptible line during active colonization (Zhou et al. 2022; Sukarta et al. 2020). Together, these results indicate coordinated but allele-specific regulatory dynamics at the *Rdm3* locus. Future studies integrating long-read sequencing, transcriptomic analyses, and functional validation will be required to resolve the genetic structure and molecular mechanisms underlying *Rdm3*-mediated resistance (Meyer et al. 2017).

### Transmission distortion and origin of the *Rdm3_Crockett* allele

Significant segregation distortion at the *Rdm3* locus was consistently observed across multiple Crockett-derived populations, resulting in underrepresentation of the *Rdm3_Crockett* resistance allele. This pattern was not observed in populations derived from other *Rdm3* sources, such as Tracy-M and PI 398469, indicating haplotype-specific differences in transmission. Consistent with this, the *Rdm3_Crockett* allele occurs at low frequency in diverse germplasm, including Brazilian soybean lines (Silva et al. 2025), elite southern breeding lines (Widyawan et al. 2026), and the USDA Soybean Germplasm Collection (Song et al. 2015), where it is present at ~0.8%.

This underrepresentation suggests that the *Rdm3_Crockett* allele may depend on additional genetic factors for stable transmission or function. Similar allele-specific dependencies have been reported at other soybean resistance loci, such as the NSF-dependent *rhg1* system for SCN resistance, where incompatible allele combinations lead to segregation distortion or reduced viability (Bayless et al. 2019; Usovsky et al. 2025). Together, these observations suggest that segregation distortion, linkage to deleterious variants, or fitness-related constraints may contribute to the low frequency of the *Rdm3_Crockett* allele.

Pedigree analysis showed that the *Rdm3_Crockett* allele is absent from Crockett’s documented parents, grandparents, and North American ancestral lines (Gizlice et al. 1994). Although SSC resistance in Crockett is likely derived from its resistant parent PI 171451, both SoySNP50K and WGRS data indicate that the *Rdm3* region in Crockett is genetically distinct from that of PI 171451. PI 171451 shares the *Rdm3* haplotype with another resistant source, Tracy-M (CGC), whereas Crockett carries a distinct haplotype defined by the nonsynonymous variant tagged by GSM1402 (CGT).

Within the Crockett lineage, Majos and S-100 exhibit *Rdm3* haplotypes corresponding to *Rdm3_PI 398469* (CTC) and a non-resistance haplotype (TGC), respectively; both are inconsistent with those of their documented parents, indicating additional complexity in inheritance. These discrepancies may reflect limitations in pedigree records or the inability of available marker data to fully capture recombination and allele transmission at this locus, despite these haplotypes being present in broader North American germplasm.

Structural variation analysis revealed substantial genomic complexity within the *Rdm3* interval, with dense clusters of breakend (BND) signals near GSM1402 in *Glyma.14G023500* across Crockett and its parental lines, indicating a structurally dynamic region. Crockett also exhibited additional local rearrangements beyond the parental background. Notably, *Glyma.14G023500* shares strong sequence homology (93% identity; BLAST E-value = 0) with a paralogous gene on Chr 2, *Glyma.02G0291300*, which also encodes a CDPK. This supports a model of non-allelic homologous recombination (NAHR), in which recombination between paralogous sequences generates novel genomic configurations (Parks et al. 2015).

Such structural rearrangements provide a plausible explanation for the origin of the distinct *Rdm3_Crockett* allele, which cannot be explained by simple Mendelian inheritance. Although rare, such events can generate alleles with adaptive advantages, including enhanced disease resistance (Liu et al. 2025). The unique *Rdm3_Crockett* allele may contribute to the broader resistance spectrum observed in Crockett compared with other *Rdm3* sources (Brumer et al. 2018), and its absence in ancestral germplasm supports a de novo origin during breeding. Future studies integrating long-read sequencing to resolve structural variants and breakpoint junctions, along with copy-number analysis and allele-specific expression profiling of *Glyma.14G023500* and its Chr 2 paralog, will be needed to clarify the structural architecture and biological impact of this allele (Cook et al. 2014; Quiroz et al. 2023; Asselin et al. 2025).

### Allelic relationships at the Rdm3 locus in additional resistance sources

Recently, Widyawan et al. (2026) identified the *Rdm3* Tracy-M-type as a major source of SSC resistance in elite southern soybean germplasm. Comparison of SoySNP50K genotypes revealed variation within the *Rdm3* interval in Tracy-M relative to Crockett and PI 398469, suggesting a distinct resistance allele. Consistent with this, Tracy-M exhibits resistance responses similar to Crockett but differs from PI 398469, as both Tracy-M and Crockett are resistant to all South American isolates tested (Brumer et al. 2018). Differences in allele transmission patterns among these lines further support allelic differentiation at this locus. Although a formal allelism test was beyond the scope of this study, integrating marker-assisted progeny analysis, sequence variation, and transcriptional profiling would provide a practical approach to resolve the allelic identity of the Tracy-M *Rdm3* locus.

## Conclusion

The *Rdm3* locus represents a major source of resistance to SSC across diverse soybean germplasm, with resistance alleles previously identified in Crockett, PI 398469, and Tracy-M. In this study, integration of progeny analysis from Crockett × PI 398469 recombinant inbred lines (RILs), sequence variation, and gene expression profiling of prioritized candidate genes clarified the allelic relationship at the *Rdm3* locus from Crockett and PI 398469. Recombinant lines carrying non-parental haplotype combinations exhibited weaker and more variable resistance than either parent, indicating disruption of favorable allele combinations. Consistent with this, sequence analysis of *Glyma.14G023500* and *Glyma.14G022600* revealed distinct amino acid variants between Crockett and PI 398469, as well as among resistant and susceptible RILs, supporting the presence of functionally distinct alleles at *Rdm3*. Notably, variation in *Glyma.14G023500* showed a more consistent association with SSC phenotypes, particularly within the protein kinase domain, suggesting that these variants contribute to the emergence of unexpected resistance phenotypes. Gene expression analysis further revealed genotype-specific temporal patterns, suggesting allele-specific regulatory mechanisms. In addition, resistance alleles from Crockett and PI 398469 differed in transmission behavior, with consistent segregation distortion and underrepresentation of the Crockett allele observed across multiple populations and germplasm pools. The Crockett allele also exhibited an atypical origin, further supporting its distinct genetic basis. These findings confirm allelic diversity at the *Rdm3* locus and provide guidance for the effective use of *Rdm3* resistance in soybean breeding.

## Electronic supplementary material

Below is the link to the electronic supplementary material.

Supplementary file 1 (DOCX 19 kb)

## Data Availability

No datasets were generated or analyzed during the current study.
