# Supplementary material for: Allele variation at the Rdm3 locus conferring resistance to soybean southern stem canker
Source: Theor Appl Genet. 2026 Sep 27;139(10):282. doi: 10.1007/s00122-026-05381-9 (PMC13616858; doi:10.1007/s00122-026-05381-9)
Supplement: Supplementary file 1 [file 122_2026_5381_MOESM1_ESM.docx]

**Supplementary Table 1.** List of KASP and qRT-PCR primers used in this study

| **No.** | **Marker name** | **Assay type** | **Chr^1^** | **Position^2^** | **Forward primer sequence**  **(5' - 3')** | **Reverse primer sequence**  **(5' - 3')** |
| --- | --- | --- | --- | --- | --- | --- |
| 1 | GSM1395 | KASP | 14 | 1,630,331 | TGTCCCTCCAAGGCAGATCTAGCT[C/T] | CTTGGCATGTAGTCATCCTCATCCTT |
| 2 | GSM933 | KASP | 14 | 1,684,178 | AGACTTGGCCTGGTTGGA[T/G] | AGACTTGGCCTGGTTGGAT |
| 3 | GSM1402 | KASP | 14 | 1,698,329 (RC) | GGTGTGTACAACAATCACAAGA[G/A] | GCCTGAGGAGGAGCATGTTTCT |
| 4 | GSM1455  (*Glyma14G.023500*) | qPCR | 14 | 1,694,041-1,699,397 | GGAGGTGCTGGTGTGTACAACA | GCCTGAGGAGGAGCATGTTTC |
| 5 | GSM1452 (*Glyma14G.022600*) | qPCR | 14 | 1,630,254-1,637,800 | TTACCTCAGGTCCAACTCATGC | AGAAAGCATTCCCCTCCAGAAG |
| 6 | GSM1110/*Cons7*  (internal control gene) | qPCR | 3 | 38,644,029-38,660,685 | ATGAATGACGGTTCCCATGTA | GGCATTAAGGCAGCTCACTCT |

^1^ Chromosome

^2^ Position based on Williams 82 version 6 (Wm82.a6) reference genome; RC = designed based on reverse complement

**Supplementary Table 2.** Selected resistant and susceptible RIL derived from Crockett × PI 398469 used for WGRS

| **No.** | **ID** | **Category** | **SSC rating** |
| --- | --- | --- | --- |
| 1 | R-RIL-025 | Resistant (Recombinant 1) | 1.4 |
| 2 | R-RIL-063 | Resistant (Recombinant 1) | 1.1 |
| 3 | S-RIL-005 | Susceptible (Recombinant 2) | 2.6 |
| 4 | S-RIL-209 | Susceptible (Recombinant 2) | 2.6 |

**Supplementary Table 3.** Soybean lines used for qRT-PCR analysis

| **No.** | **Line** | **Pedigree** | **Phenotype^1^** |
| --- | --- | --- | --- |
| 1 | Crockett | Parental line | Resistant |
| 2 | PI 398469 | Parental line | Resistant |
| 3 | G81-2057 | Parental line | Susceptible |
| 4 | G19-2950 | F_5:6_ G81-2057 × Crockett | Resistant (Crockett-derived) |
| 5 | G19-3059 | F_5:6_ G81-2057 × Crockett | Susceptible (Crockett-derived) |
| 6 | G19-1860 | F_5:6_ G81-2057 × PI 398469 | Resistant (PI 398469-derived) |
| 7 | G19-1857 | F_5:6_ G81-2057 × PI 398469 | Susceptible (PI 398469-derived) |

^1^ Southern stem canker (SSC) resistance phenotype according to Menke et al. (2023)

**Supplementary Table 4.** Chi-square tests for segregation distortion using GSM1402 and GSM1395 across populations segregating for *Rdm3* locus (expected 1:1 ratio)

| **Marker** | **Population** | **Observed^1^ (Res:Sus)** | ***χ²*** | **P-value** | **Segregation pattern** |
| --- | --- | --- | --- | --- | --- |
| GSM1402 | Crockett × PI 398469 (F_2_) | 120:156 | 4.7 | 0.03 | Significant distortion |
|  | Crockett × PI 398469 (F_2:3_) | 78:194 | 49.5 | <0.0001 | Significant distortion |
|  | Crockett × PI 398469 (RIL) | 96:188 | 29.8 | <0.0001 | Significant distortion |
|  | Tracy-M × Crockett (F_2:3_) | 102:163 | 14 | <0.0001 | Significant distortion |
|  | G81-2057 × Crockett (RIL) | 56:90 | 7.9 | 0.005 | Significant distortion |
| GSM1395 | G00-3213 × Tracy-M (F_2:3_) | 68:68 | 0 | 1 | No distortion |
|  | G81-2057 × PI 398469 (F_2:3_) | 69:83 | 1.3 | 0.26 | No distortion |
|  | G81-2057 × PI 398469 (RIL) | 107:101 | 0.2 | 0.68 | No distortion |
|  | G81-2057 × Crockett (RIL) | 65:93 | 4.96 | 0.05 | Significant distortion |

^1^ Res = resistance allele; Sus = susceptible allele
